# Advancing Genitourinary Cancer Surgery: The Role of Artificial Intelligence and Robotics

**DOI:** 10.3390/jcm15103856

**Published:** 2026-05-17

**Authors:** Stamatios Katsimperis, Nikolaos Kostakopoulos, Themistoklis Bellos, Theodoros Spinos, Angelis Peteinaris, Lazaros Tzelves, Athanasios Kostakopoulos, Andreas Skolarikos

**Affiliations:** 1 Second Department of Urology, National and Kapodistrian University of Athens, Sismanogleio Hospital, 15126 Athens, Greece; 2 First Department of Urology, Metropolitan General Hospital, 15562 Athens, Greece; kostako@doctor.com; 3 Third Department of Urology, Mitera Hospital, 15123 Athens, Greece; 4 Department of Urology, University of Patras Hospital, 26504 Patras, Greece; thspinos@otenet.gr (T.S.);

**Keywords:** artificial intelligence, robotic-assisted surgery, genitourinary oncology, prostate cancer, renal cell carcinoma, bladder cancer, augmented reality, intraoperative navigation, single-port robotic platforms, telesurgical systems

## Abstract

The convergence of artificial intelligence and robotic surgery is redefining the management of genitourinary cancers by enhancing diagnostic accuracy, surgical precision, and training efficiency. This narrative review explores recent advancements in artificial intelligence applications across the cancer care continuum, with a focus on prostate, kidney, and bladder malignancies. Artificial intelligence tools, particularly those based on machine learning and deep learning, have demonstrated strong performance in analyzing imaging data, segmenting tumors, predicting pathological features, and supporting clinical decision-making. Intraoperatively, artificial intelligence enables skill assessment, personalized feedback, and real-time navigation by processing data from surgical videos and robotic system sensors. Augmented reality and intraoperative modeling further enhance visualization and margin control during complex procedures. The review also discusses emerging technologies such as single-port robotic platforms, which offer advantages in confined anatomical spaces and support less invasive approaches. Additionally, the growing field of telesurgery is addressed, highlighting its feasibility for complex urologic operations across vast distances. While many of these innovations are still in early stages of clinical validation, their integration into practice has the potential to improve oncologic and functional outcomes, expand access to expert care, and foster the development of next-generation surgical strategies in urologic oncology.

## 1. Introduction

The landscape of genitourinary (GU) oncology has been transformed over the past two decades by two synergistic technological frontiers: robotic-assisted surgery and artificial intelligence (AI) [1,2]. While robotic platforms have redefined the precision, dexterity, and ergonomics of minimally invasive urologic surgery, the rapid integration of AI has ushered in a new era of intelligent surgical systems that extend human capabilities beyond mechanical enhancement. In this evolving paradigm, surgeons are no longer just operators of machines, but active participants in a digitally augmented surgical ecosystem powered by real-time data, machine learning algorithms, and computational vision. Robot-assisted surgery, initially dominated by the da Vinci multi-arm platform, has become standard in procedures such as radical prostatectomy (RARP), partial nephrectomy (RAPN), and radical cystectomy (RARC). These systems offer three-dimensional visualization, articulated instrument control, and tremor filtration, facilitating delicate dissections in anatomically complex and confined regions. However, despite their mechanical superiority, conventional robotic systems rely entirely on the surgeon’s cognitive and visual processing. This is where artificial intelligence becomes a transformative adjunct. AI, particularly through its subfields of machine learning (ML), deep learning (DL), and computer vision (CV), offers new capabilities in pattern recognition, data integration, and predictive analytics. In urologic oncology, AI applications are already proving useful across the perioperative continuum [3,4]. Preoperatively, AI enables improved radiologic interpretation, risk stratification, and surgical planning [5]. Intraoperatively, it enhances visualization, anatomical mapping, and tool guidance. Postoperatively, it contributes to outcome prediction, complication monitoring, and personalized follow-up protocols. The integration of AI into robotic systems is also producing a new generation of intelligent surgical assistants. These tools are capable of recognizing procedural phases, analyzing instrument movement, providing autonomous feedback, and even proposing corrective actions in real-time. In the training realm, AI-driven simulators and automated performance metrics (APMs) are revolutionizing how surgical competence is assessed and acquired [6]. Moreover, innovations such as single-port (SP) robotic systems, 5G-enabled telesurgery, and AI-powered augmented reality (AR) overlays are expanding the scope and reach of robot-assisted interventions [7,8]. In this digitally connected framework, surgeons can perform remote operations, visualize anatomy with dynamic overlays, and consult intelligent guidance systems throughout complex procedures. However, these advances do not come without challenges. The validation, regulation, and ethical implementation of AI tools in surgical oncology require robust frameworks to ensure safety, equity, and transparency. Issues such as data bias, algorithm interpretability, and shared liability in AI-augmented decision-making are under active debate. Furthermore, cost and access disparities may widen without deliberate strategies to broaden access to these technologies.

This narrative review aims to explore the intersection of AI and robotic surgery in the management of GU cancers. We synthesize current evidence on the clinical integration of AI in surgical planning, real-time intraoperative support, postoperative outcome prediction, and surgical education. Drawing on developments in prostate, kidney, and bladder cancer surgery, we highlight the technologies shaping the future of precision oncology and consider the implications for surgeons, institutions, and patients alike.

## 2. Methods

This narrative review was conducted through a comprehensive literature search of PubMed/MEDLINE, Scopus, and Web of Science databases, covering publications from inception to November 2025. Search terms included combinations of ‘artificial intelligence’, ‘machine learning’, ‘deep learning’, ‘robotic surgery’, ‘robot-assisted’, ‘prostate cancer’, ‘renal cell carcinoma’, ‘bladder cancer’, ‘augmented reality’, ‘surgical navigation’, ‘single-port robotic’, and ‘telesurgery.’ Studies were selected based on their relevance to AI applications in robotic genitourinary oncology surgery, prioritizing peer-reviewed original research, prospective and retrospective clinical studies, and key systematic reviews or meta-analyses. Case reports with fewer than five patients, conference abstracts without full-text availability, and non-English-language publications were excluded. Reference lists of included articles were also screened to identify additional relevant studies. The final selection was agreed upon by consensus among the authors. Given the narrative nature of this review, a formal systematic screening process with predefined inclusion and exclusion criteria was not performed. The included studies were selected to provide a comprehensive and representative, rather than exhaustive, overview of the current evidence on AI and robotic technologies in genitourinary cancer surgery.

## 3. AI Applications in Robotic Genitourinary Oncology

### 3.1. AI-Enhanced Preoperative Planning: Imaging Analysis and Tumor Segmentation

AI-based imaging models applied to MRI, CT, and PET are increasingly being investigated in urologic oncology for tumor identification, localization, segmentation, and characterization, with mpMRI-based convolutional neural networks showing encouraging performance in detecting clinically significant prostate cancer lesions [9,10,11]. AI-based radiomics approaches have also been applied to correlate imaging phenotypes with Gleason score, extracapsular extension, and lymph node involvement [12]. These insights are used to create predictive models that support decision-making regarding nerve-sparing approaches or extended pelvic lymph node dissection during RARP. In a retrospective, single-center study by Takeuchi et al., researchers investigated the potential of using a multilayer artificial neural network (ANN) to predict prostate cancer based on multiparametric MRI in patients undergoing biopsy, using histopathological biopsy results as the reference standard [13]. The study included 334 participants, with 232 allocated to the training set and 102 to the testing set. Among the patients without prostate cancer, 48% were evaluated [13]. The ANN failed to detect 16% of prostate cancer cases with a Gleason score of 7 or higher, and 6% of high-grade cases were also missed. In a related effort, Seetharaman et al. developed a CNN model to identify clinically significant prostate cancer using MRI data in a retrospective analysis [14]. When tested on patients who underwent fusion prostate biopsy and prostatectomy, with whole-mount histopathology serving as the gold standard, the model achieved area under the curve (AUC) values of 75% and 80%, respectively [14]. Notably, it also identified 18% of cancerous lesions that had been missed by radiologists, highlighting its potential to complement human interpretation. Additional deep learning studies have also explored automated prostate segmentation, lesion localization, and PI-RADS-like stratification, with the aim of reducing inter-reader variability [15]. Early investigations also demonstrated that combining MRI data with histopathological inputs can enhance the accuracy of computer-aided diagnostic systems [16]. Radiomics-based analyses further extend this approach by extracting quantitative features from T2-weighted and diffusion-weighted sequences to predict clinically relevant factors such as Gleason grade, extracapsular extension, and recurrence risk. When combined with clinical variables such as PSA density and digital rectal examination findings, mpMRI radiomics models have reported AUCs of approximately 0.84–0.89 for clinically significant prostate cancer detection, suggesting potential value for diagnostic decision-making and surgical planning [17,18]. It should be noted that the majority of these CNN and radiomics-based models remain research tools developed and validated on institutional datasets. To date, none have received regulatory clearance (e.g., FDA or CE marking) for routine clinical use in prostate cancer diagnosis or surgical planning, although several commercial AI-assisted MRI interpretation platforms are under development or in early clinical deployment outside the urologic oncology context.

In renal cancer, AI tools applied to contrast-enhanced CT or MRI scans can distinguish benign renal tumors from renal cell carcinoma (RCC), classify renal masses (e.g., clear cell vs. non-clear cell RCC), and estimate tumor complexity (e.g., RENAL nephrometry scores). In a retrospective, multicenter study by Xi et al., a deep learning approach was employed to differentiate renal cell carcinoma from benign kidney tumors using magnetic resonance imaging data [19]. The model analyzed a dataset of 1162 renal lesions and incorporated both imaging features and clinical information through an ensemble architecture based on ResNet [19]. Compared to expert radiological assessments and the top-performing radiomics model, the DL framework yielded superior diagnostic metrics, including an accuracy of 0.70. In a related effort to characterize renal tumor subtypes, a radiomics-based machine learning model was developed by Budai et al. to differentiate clear cell RCC from non-clear cell variants using contrast-enhanced CT scans [20]. The model was trained and validated on multiphase CT scans, primarily utilizing features from the corticomedullary phase after filtering for reproducibility and relevance. When externally validated on an independent dataset, it demonstrated an AUC of 0.83 and performance metrics comparable to those of an expert radiologist [20]. In the context of differentiating renal tumors, Coy et al. applied a deep learning model to distinguish between clear cell renal cell carcinoma and oncocytoma in a cohort of 179 patients with renal masses, achieving a classification accuracy of 74.4% [21]. Such predictions can inform personalized treatment strategies, guiding decisions between partial or radical nephrectomy as well as the selection of systemic therapies in advanced or high-risk cases. These deep learning and radiomics models are currently at the research stage and have not been integrated into commercially available diagnostic platforms for renal mass characterization.

For bladder cancer, AI models are being developed to detect muscle invasiveness from MRI and CT sequences using deep segmentation networks. These models can distinguish between NMIBC and MIBC, offering non-invasive support for staging decisions. Earlier studies demonstrated promising outcomes: Yang et al. analyzed 1200 CT images using eight different deep learning algorithms, with one model achieving an AUC of 99.8% for NMIBC vs. MIBC classification [22]. Similarly, Xu et al. used 1104 MRI images to develop an AI model that outperformed conventional assessments, achieving an accuracy of 96.3% and an AUC of 98.6% [23]. Building on this, Sarkar et al. employed a hybrid deep learning model to classify bladder cancer using a series of axial CT scans with intravenous contrast [24]. The model achieved its highest accuracy, over 86%, in detecting bladder cancer versus normal tissue, while NMIBC versus MIBC classification reached nearly 80% [24]. Although tissue biopsy remains the gold standard, these AI models may enhance clinical decision-making by offering early, non-invasive insights into tumor invasiveness, aiding in patient triage and preoperative staging. Beyond staging, recent efforts have explored AI’s role in preoperative tumor grading. Beyond staging, CT-based deep learning radiomics nomograms have also been explored for preoperative tumor grading, including differentiation between high- and low-grade bladder cancer [25]. As with prostate and renal applications, these bladder cancer AI models remain investigational and have not yet been incorporated into commercially available clinical tools. Prospective validation in multicenter settings is needed before clinical translation.

Across the diagnostic imaging studies reviewed here, an important limitation is that most reported performance metrics derive from retrospective datasets, frequently from single-center or highly selected cohorts. Moreover, the reference standards are not uniform across studies and include targeted or systematic biopsy results, radical prostatectomy specimens, postoperative histopathology, expert radiologic annotation, or composite clinical staging endpoints. This heterogeneity should be considered when interpreting reported sensitivity, specificity, accuracy, or AUC values, and it underscores the need for prospective multicenter validation before broad clinical implementation.

### 3.2. AI in Surgical Skill Assessment and Performance Optimization

Artificial intelligence is increasingly being applied to evaluate surgeon performance in robot-assisted genitourinary procedures. Modern robotic platforms generate detailed data, such as instrument motion and system event logs, that can be analyzed with machine learning to assess efficiency, dexterity, and procedural flow. Deep learning models, including CNNs, RNNs, and transformers, are also used to detect subtle inefficiencies or patterns in movement, enabling objective feedback and long-term performance monitoring that may go beyond traditional peer review.

AI-based video analysis tools have further advanced surgical training and evaluation. For example, the Video Analysis of Skill and Technique (VAST) system has been used to assess bladder neck anastomosis by analyzing console video feeds from 11 surgeons [2]. The system achieved 83% accuracy with single-instrument data, 92% with bilateral instruments, and 100% when incorporating contralateral movement, validated against expert GEARS scoring [2]. Another application was reported by Youssef et al., who developed an AI tool to assess RARP procedures using the Proximie AR platform (v0.16.1) [26]. In this study, a novice surgeon annotated the procedural steps of 25 RARP videos. Of these, 17 were suitable for analysis, with the model achieving temporal tagging accuracy between 85.6% and 100%, averaging 93.1% [27]. In parallel, Hung et al. developed a computer vision-based framework to automate the assessment of suturing skills using video and kinematic data from robotic surgery simulations [28]. The study analyzed 226 virtual sutures performed by 22 surgeons, segmenting each stitch into standardized sub-tasks and scoring performance domains such as needle positioning, entry, and withdrawal. Their system extracted visual cues from the red–green–blue (RGB) video channels along with optical flow data to capture motion dynamics. When combined with advanced techniques such as auxiliary supervision and attention mechanisms, the model achieved high predictive performance—particularly for needle driving and withdrawal, with AUCs of 0.959 and 0.879, respectively [28]. These results highlight the growing capability of AI to evaluate fine motor skills objectively, supporting its role in surgical education and performance feedback. Building on this concept, a pilot study by Ma et al. investigated whether AI-based video feedback could improve novice performance in robotic suturing [29]. Forty-two participants with no prior robotic experience were randomized to receive either algorithm-driven visual feedback or placebo video review [29]. The group receiving AI feedback showed significantly greater improvement in needle handling scores, particularly among underperformers. These findings suggest that AI-powered assessment systems not only measure skill but can also accelerate learning by delivering personalized, actionable insights. It is important to note that these AI-based assessment tools, including the VAST system and the Proximie-based annotation framework, are research platforms and are not yet integrated as standard features within commercial robotic consoles. Current robotic systems, including the da Vinci Xi and SP, do not offer built-in AI-driven skill assessment, although the da Vinci 5 platform incorporates enhanced data-capture capabilities that may facilitate such integration in the future.

In addition to supporting individual feedback and coaching, AI-derived performance data can be aggregated across institutions to help establish benchmarks for surgical proficiency and identify areas for improvement. Incorporating such tools into simulation-based training or credentialing programs could contribute to more standardized and objective skill assessment. As the field evolves, real-time feedback systems may eventually be integrated into robotic consoles to further enhance intraoperative awareness and surgical education.

## 4. AI-Enhanced Imaging, Augmented Reality, and Intraoperative Navigation

Advances in AI are increasingly enhancing both the planning and execution of complex genitourinary surgeries. From integrating detailed imaging into surgical navigation systems to enabling real-time tissue recognition, AI technologies are creating new opportunities for precision, safety, and efficiency in the operating room.

### 4.1. Preoperative Imaging Integration and Augmented Reality in Surgical Planning

**Figure 1:**
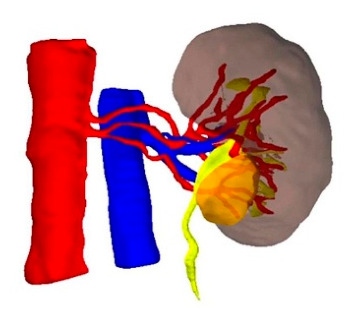
Three-dimensional virtual anatomical model used for preoperative planning and intraoperative guidance during robotic partial nephrectomy.

AI enables the transformation of preoperative imaging—such as contrast-enhanced CT or multiparametric MRI—into actionable, three-dimensional visual maps and models (3DVMs). These 3DVMs are integrated into robotic platforms using AR to support anatomical orientation and surgical planning. The combination of AI and AR allows surgeons to visualize tumor location, vascular structures, and surrounding anatomy in a dynamic, intuitive format (Figure 1). In partial nephrectomy, AI-enhanced three-dimensional (3D) reconstructions help define tumor margins, identify segmental vasculature, and support nephron-sparing strategies. Recent studies have highlighted the value of integrating 3DVMs into the preoperative planning of partial nephrectomy, particularly for improving visualization of the renal vasculature [30]. This enhanced anatomical insight supports more refined clamping techniques, such as selective or super-selective arterial clamping, which are crucial for limiting warm ischemia and preserving postoperative renal function. A notable advancement includes perfusion-region 3DVMs, which apply Voronoi-based algorithms to map distinct perfusion zones within the kidney. In a prospective cohort of over 100 robot-assisted partial nephrectomies, these perfusion-based models helped guide individualized vascular control, resulting in strong alignment between surgical planning and intraoperative execution [31]. Among the AR platforms discussed, the Hyper Accuracy 3D (HA3D^®^) system by Medics^®^ (Turin, Italy) is a commercially available product that has been used in clinical settings since approximately 2018. In contrast, perfusion-region 3DVMs and Voronoi-based algorithms remain research-level tools not yet available as standalone commercial products. Functional outcomes, measured through postoperative scintigraphy, were notably improved in cases where selective clamping was utilized [31].

### 4.2. Augmented Reality for Surgical Navigation in Urologic Oncology

AR-based navigation integrates preoperative 3D reconstructions with the live robotic endoscopic view, aiming to improve intraoperative orientation during complex urologic oncology procedures.

**Figure 2:**
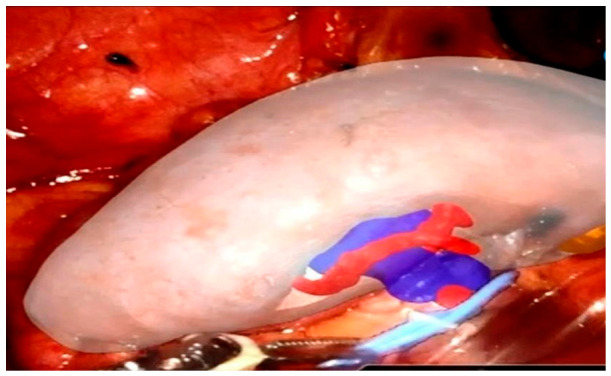
Augmented Reality in RAPN. Real-time projection of tumors and vessels onto the kidney during surgery.

In RAPN, AR has been particularly valuable in guiding the resection of complex renal tumors. By projecting critical elements such as tumor margins, collecting systems, and segmental vasculature directly onto the kidney surface in the robotic console, AR enhances intraoperative decision-making (Figure 2). Compared with traditional imaging methods such as 2D intraoperative ultrasound, AR-based navigation has shown improved performance in localizing deep or endophytic tumors and in achieving nephron-sparing strategies [32]. In the study by Porpiglia et al., HA3D^®^-guided RAPN was associated with more frequent selective or super-selective clamping, higher tumor enucleation rates, fewer collecting system violations, and better short-term renal functional preservation compared with standard guidance [32]. In parallel, Kobayashi and colleagues introduced a navigation platform fusing real-time endoscopic views with preoperative 3D models [33]. Follow-up analysis confirmed improved parenchymal preservation (90.0% vs. 83.5%, *p* = 0.042) and reduced extraparenchymal resection compared to conventional techniques (21.4 mL vs. 17.2 mL, *p* = 0.041) [34].

More recently, the integration of AI into AR platforms has further streamlined their use. The iKidney system has explored automated intraoperative alignment of 3D kidney models using deep learning-based image recognition, reducing the need for manual registration [35]. The iKidney system remains a research prototype and is not commercially available. By contrast, HA3D^®^ has been clinically deployed in selected centers. More recent work has evaluated AI-assisted AR registration in minimally invasive partial nephrectomy. In a 105-patient study, an AI-driven system matched CT-derived 3D vascular models to the surgical field using limited calibration landmarks and dynamically adjusted the overlay during kidney manipulation [36]. This approach was associated with reduced renal hilum exposure time without increasing warm ischemia time, blood loss, or postoperative complications, although broader validation is still required [36]. Although this framework was developed for laparoscopic and multi-port robotic approaches, similar AI-assisted AR concepts may be relevant to SP-RAPN, where restricted triangulation and reduced instrument spacing make orientation more challenging.

In robotic prostatectomy, AR-guided fusion of mpMRI-derived 3D models with the endoscopic view may help localize tumor foci and suspected extracapsular extension during nerve-sparing and dissection phases. For instance, use of HA3D^®^ platforms has shown promising results, with some studies reporting complete concordance between AR-targeted lesions and postoperative histopathology, and targeted biopsies confirming ECE in over 70% of cases [37]. Validation studies of these models have demonstrated high anatomical fidelity, with the majority of mapped prostate surfaces showing deviations of less than 3 mm when compared to postoperative specimens [38]. To improve adaptability to tissue deformation, elastic 3D models have been developed, offering dynamic simulation of anatomical shifts during manipulation. When tested in comparative settings, such tools have achieved full accuracy in identifying capsular involvement, outperforming traditional 2D visual estimation techniques, which demonstrated significantly lower sensitivity (47%, *p* < 0.05) [39]. These innovations suggest that AR integration may help reduce surgical margin positivity and preserve functional outcomes in patients undergoing RARP.

AI-assisted analysis of PSMA PET-CT and MRI-derived tumor maps has also been explored for margin-conscious planning and projection of suspected extracapsular extension zones during prostatectomy [40,41]. However, these systems should be regarded as adjuncts to intraoperative judgment rather than direct real-time margin detection tools.

Despite these promising findings, AR-guided robotic navigation remains at an early stage of clinical translation. Most studies are retrospective, involve limited sample sizes, or originate from highly specialized centers with substantial expertise in 3D reconstruction and robotic surgery. In addition, registration accuracy may be affected by tissue deformation, organ mobilization, bleeding, and differences between preoperative imaging and intraoperative anatomy. These limitations should be considered before extrapolating reported benefits to routine practice.

### 4.3. Immersive Surgical Planning and Virtual Collaboration

Advancements in virtual reality (VR) and connectivity technologies have enabled the emergence of immersive, collaborative environments for surgical planning. Sometimes described as part of the evolving “surgical metaverse,” these platforms allow multiple participants—surgeons, radiologists, and trainees—to interact with hyper-detailed 3D models in real time using VR headsets. Within these virtual spaces, teams can review patient-specific anatomy, simulate resection or clamping strategies, and collectively refine surgical plans before entering the operating room. A recent application of this approach by Checcucci et al. involved simulated planning for robotic partial nephrectomy, where participants convened in a virtual room to assess 3D reconstructions of renal tumors and vascular anatomy [42]. This interactive setting enhanced spatial understanding and facilitated consensus on complex surgical decisions, regardless of the physical location of the contributors. While still in early development, such immersive platforms may improve preoperative preparation, promote team-based planning, and ultimately lead to more precise and confident surgical execution.

## 5. Single-Port Robotic Surgery and Telesurgery in Urologic Oncology

### 5.1. Single-Port Robotic Surgery

SP robotic systems represent a next-generation evolution in minimally invasive surgery, aiming to reduce access trauma, improve cosmetic outcomes, and minimize recovery time by consolidating all instruments and the endoscopic camera into a single entry point. Beyond minimizing surgical trauma, this design offers superior instrument control in confined anatomical spaces, making it especially advantageous in urologic oncology, where target regions are often deep and narrow. In urologic oncology, SP platforms—such as the da Vinci SP system—have been adopted for a growing range of procedures including radical prostatectomy, partial nephrectomy, and radical cystectomy. These applications have demonstrated technical feasibility, with early series reporting favorable operative times, acceptable complication rates, and satisfactory oncologic outcomes in selected patient populations.

Prostate cancer remains the most widely studied domain for SP robotic surgery in urologic oncology. SP robot-assisted radical prostatectomy (SP-RARP) has gained momentum as an effective surgical option for localized disease, combining minimal invasiveness with promising perioperative outcomes. Since the initial reports by Kaouk et al. demonstrating the feasibility of SP-RARP with low complication rates and early discharge, interest in this approach has grown substantially [7,43]. Multiple surgical access routes, such as transperitoneal, extraperitoneal, Retzius-sparing, and transvesical, have been successfully adapted to the SP platform, each offering specific technical and recovery-related benefits [44,45,46,47]. Supporting these outcomes, Abaza et al. reported same-day discharge in 88% of their SP-RARP patients, attributing this to reduced postoperative pain and quicker recovery associated with the single-incision design [48]. Similarly, Vigneswaran et al. observed lower pain scores on the first postoperative day in patients undergoing SP-RARP compared to multi-port (MP) approaches, reinforcing the potential of SP surgery to enhance early convalescence [49]. Reduced blood loss has also been reported with SP-RARP compared to MP-RARP; however, the difference—typically around 50 mL—is modest, and its clinical relevance remains uncertain [50,51,52]. Regarding urinary continence and erectile function, most studies indicate parity between SP and MP platforms at three to six months postoperatively [45,53,54]. For partial nephrectomy, the SP system offers a unique advantage in retroperitoneal access due to its compact design, especially in posteriorly located renal tumors. However, current literature has not demonstrated a clear oncologic benefit over multi-port approaches [55,56]. Notably, some studies have reported longer warm ischemia times (WIT) in the SP-RAPN group compared to MP-RAPN, potentially reflecting the early learning curve and technical challenges associated with the single-port platform [55,57,58]. SP robot-assisted radical cystectomy (SP-RARC) remains an emerging application within urologic oncology, primarily due to the complexity and multi-quadrant nature of the procedure. Unlike prostatectomy or partial nephrectomy, cystectomy involves extended lymphadenectomy and urinary diversion, which pose significant technical demands. As a result, clinical data on SP-RARC remain limited but are steadily growing. In the largest series to date comparing SP to MP RARC, Fang et al. evaluated 96 patients and found that SP-RARC was associated with shorter operative time (386 vs. 454 min, *p* < 0.01) and quicker bowel recovery (3.4 vs. 4.5 days, *p* < 0.01), while complication rates, readmissions, and positive margin rates remained comparable between groups [59].

Despite these promising applications, the transition to SP surgery is not without significant technical and ergonomic challenges. The most notable difference from MP systems is the loss of natural triangulation, as SP instruments work through a single axis of entry. Although the system compensates with wristed instruments and a flexible camera, this requires new visuospatial skills and a period of familiarization. The learning curve for SP robotic surgery is considerable, even for surgeons already proficient in MP techniques [60]. Early adopters report that complex tasks such as renorrhaphy during partial nephrectomy or bladder reconstruction in cystectomy demand a high degree of technical adaptation. As a result, stepwise training protocols, including simulation, dry-lab exercises, and mentorship, are being developed to support the safe implementation of SP systems into surgical practice. Moreover, instrument limitations currently restrict the scope of SP procedures. The unavailability of key tools such as the ProGrasp™ forceps, robotic staplers, and Firefly^®^ near-infrared fluorescence imaging constrains vascular control and reconstruction options. Until a broader armamentarium is integrated into the SP system, some procedures may still favor MP approaches for comprehensive oncologic resection and reconstruction. Port placement and workspace constraints also play a critical role in the success of SP surgery. A minimal distance—generally at least 10 cm—is required between the SP cannula and the target organ to allow for adequate instrument articulation. This makes SP surgery more challenging in patients with high body mass index, prior abdominal surgery, or distorted pelvic anatomy. In such cases, careful preoperative imaging and port planning are essential.

Nevertheless, SP robotic surgery has shown comparable perioperative outcomes to MP surgery in well-selected patients. These findings, along with continued hardware innovation and expanding surgical experience, support a growing role for SP platforms in minimally invasive urologic oncology. Future improvements, such as wider instrument availability, enhanced imaging integration, and AI-assisted navigation, may further expand the indications for SP surgery and reduce its technical barriers.

Beyond current single-port platforms, the newly introduced da Vinci 5 represents the next major step in robotic evolution, with advanced sensing, imaging, and data-capture capabilities that significantly expand its suitability for AI-driven model development. In addition to enhanced instrument dexterity and improved ergonomics, the fifth-generation system incorporates a redesigned vision stack with more powerful 3D processing, higher-fidelity imaging, and substantially upgraded onboard computing [61]. These innovations enable the collection of richer video streams, highly precise kinematic logs, and multimodal intraoperative data suitable for large-scale machine-learning workflows. The platform’s expanded computational capacity also lays the foundation for future AI-enabled functionalities, such as real-time workflow analysis, automated safety alerts, and context-aware assistance. Although many of these features remain in the early phases of integration, the da Vinci 5 marks an important shift toward robotic systems that are inherently designed to acquire, structure, and leverage high-quality data for next-generation surgical intelligence. The da Vinci SP system received FDA clearance in 2018 and is commercially available, with growing adoption worldwide. The da Vinci 5, introduced in 2024, is also commercially available and represents the latest generation of the Intuitive Surgical platform.

### 5.2. Telesurgery and Remote Robotics

Telesurgery, the performance of surgery by a surgeon at a distance using robotic systems, represents a compelling future direction for AI-integrated robotic platforms. By leveraging high-speed communication networks and advanced robotic control systems, telesurgery promises to transcend geographic barriers, bringing subspecialty expertise to underserved or remote regions without requiring patient transfer. Although still in its early stages of clinical adoption, proof-of-concept demonstrations and pilot trials have shown its feasibility and growing relevance in urologic oncology.

Emerging clinical evidence, particularly from China, has demonstrated the technical feasibility of both single-port and multi-port telesurgical procedures over distances surpassing 6000 km. These include complex urologic interventions such as radical prostatectomy and cystectomy, performed without intraoperative complications and with transmission latency consistently below 200 milliseconds [8,62,63]. Robotic systems like the MicroHand Edge MP1000, Toumai^®^, and KangDuo have played a key role in these advancements, offering features such as multi-console configurations, real-time surgeon collaboration, and robust latency management [64,65]. Beyond proving technical success, these developments underscore the potential of telesurgery to expand access to specialized care in remote or resource-limited settings. However, broader clinical integration will require careful attention to cybersecurity, regulatory credentialing, cost-efficiency, and the current limitations in haptic feedback. Toumai has received regulatory approvals including CE certification for its laparoscopic system and NMPA approval in China for tele-robotic applications; however, its deployment remains regionally variable. KangDuo and other Chinese robotic systems have been used in telesurgical demonstrations and selected clinical settings, but broad international availability and regulatory clearance remain limited or evolving.

Beyond technical feasibility, the safe and ethical implementation of telesurgery in urology has recently been addressed through comprehensive policy efforts. The European Association of Urology (EAU) now endorses telesurgical procedures, provided they are conducted within a structured framework that prioritizes patient safety, clinical governance, and regulatory oversight [66]. These guidelines emphasize rigorous preoperative planning, informed consent, intraoperative protocols, and postoperative care standards to ensure high-quality outcomes and minimize risk. The EAU also calls for ongoing training, data collection, and audit mechanisms to support responsible adoption and long-term sustainability of telesurgical practices across clinical and educational domains. To improve clarity and distinguish between established, emerging, and investigational applications, the main AI-enabled and robotic technologies discussed in this review are summarized in Table 1.

## 6. Limitations and Future Directions

This work carries the inherent limitations of a narrative review, including the absence of a fully reproducible search protocol and the potential for selection bias in the identification and inclusion of studies. Additionally, the majority of AI studies discussed are retrospective and single-center in design, and their reported performance metrics may not directly translate to real-world clinical settings, where data heterogeneity, workflow integration, and regulatory requirements pose additional challenges.

Despite rapid advances, several barriers remain before AI can be fully integrated into routine urologic oncology practice. One of the primary challenges is the availability and quality of annotated data. High-performing AI models require large, diverse, and well-labeled datasets to ensure generalizability and robustness. However, data heterogeneity, including differences in imaging protocols, surgical techniques, and institutional workflows, can affect model performance across centers.

There are also important ethical, legal, and regulatory concerns. Clinicians and institutions must consider data privacy, algorithmic transparency, and accountability in cases where AI influences clinical decisions. The “black box” nature of many deep learning models makes interpretability difficult, potentially limiting clinician trust and adoption. Another important direction is the incorporation of explainable AI (XAI) methods to overcome the inherent “black-box” character of many deep learning systems. Techniques such as saliency mapping and gradient-based class activation (e.g., Grad-CAM) have been applied to mpMRI prostate cancer models to visualize the specific image regions driving lesion classification, thereby improving interpretability and supporting clinician confidence [67,68,69]. In radiomics-based models, feature attribution methods such as SHAP (SHapley Additive exPlanations) can quantify the contribution of individual texture or intensity features to predictions of tumor grade or extracapsular extension. Recent machine-learning work in prostate cancer treatment selection has also employed SHAP to identify the clinical and pathological variables most strongly driving model recommendations, demonstrating how transparent feature ranking can assist clinicians in understanding and validating AI-derived decisions [70]. Similarly, attention mechanisms used in video-based models for robotic surgery provide heatmaps that highlight the instrument trajectories or tissue planes most relevant to phase recognition or skill assessment. These explainability tools not only clarify how AI systems reach their conclusions but also support ethical implementation by ensuring that surgeons remain the final decision-makers in the operative workflow. Regulatory frameworks for approving AI tools in surgery are still evolving, and clear guidelines on liability, validation, and integration into clinical workflows are needed.

Moreover, many AI applications remain in early developmental stages or limited to retrospective analyses. Prospective clinical trials, multicenter collaborations, and real-world validations are essential to demonstrate clinical benefit and cost-effectiveness. Future research should focus on integrating multimodal data—combining imaging, pathology, genomics, and surgical performance metrics—into unified predictive models that support truly personalized surgical care.

The near-term development of AI in urologic oncology surgery is likely to be shaped by the convergence of three major trends. First, the increasing availability of high-quality, multimodal surgical data, driven by platforms such as the da Vinci 5 with its enhanced data-capture capabilities, will enable the training of more robust and generalizable AI models. Second, we anticipate that AI-powered intraoperative decision support systems will gradually transition from retrospective research tools to real-time clinical assistants, offering surgeons context-aware guidance during critical procedural steps such as hilar dissection in partial nephrectomy or nerve-sparing during radical prostatectomy. Third, the maturation of federated learning approaches, where AI models are trained across multiple institutions without sharing raw patient data, may help overcome current barriers related to data privacy, institutional heterogeneity, and limited dataset sizes, ultimately producing models that generalize across diverse clinical settings.

Looking further ahead, integration of AI with next-generation robotic platforms may gradually extend beyond decision support toward partial automation of selected, well-defined surgical subtasks. While fully autonomous surgery remains a distant prospect, the progressive automation of discrete, well-defined subtasks, such as suturing, tissue retraction, or camera positioning, is a realistic medium-term goal. Additionally, the combination of AI-driven augmented reality with 5G-enabled telesurgery could potentially improve access to specialized urologic cancer care, enabling expert-guided or remotely supervised procedures in underserved regions worldwide. The expansion of single-port platforms, equipped with broader instrument portfolios and AI-enhanced navigation, may further reduce surgical invasiveness while maintaining oncologic efficacy. However, realizing this vision will require not only technological progress but also the development of robust regulatory frameworks, standardized validation pipelines, and training curricula that prepare the next generation of urologic surgeons to work effectively alongside intelligent surgical systems.

To facilitate widespread adoption, surgical teams must be equipped with training on AI fundamentals, limitations, and best practices. Cross-disciplinary collaboration among surgeons, data scientists, engineers, and ethicists will be critical in designing tools that are not only technically sound but also clinically meaningful and ethically aligned. As the field progresses, the synergy between AI and robotics holds the promise to enhance precision, improve outcomes, and transform the future of genitourinary cancer surgery.

## 7. Conclusions

Artificial intelligence is increasingly reshaping the landscape of robotic surgery in genitourinary oncology, offering tools that enhance imaging interpretation, intraoperative navigation, surgical performance assessment, and procedural planning. The integration of augmented reality, advanced deep learning models, and single-port platforms is driving a more precise, personalized, and minimally invasive approach to cancer surgery. Although many of these technologies remain under active investigation, early results are promising. Continued validation and broader clinical adoption will be essential to realize their full potential in improving outcomes and reducing disparities in urologic cancer care.

## Figures and Tables

**Table 1:** Summary of AI and Robotic Technologies in Genitourinary Cancer Surgery: Techniques, Products, Development Stage, and Clinical Benefits.

Application Domain	Technique/Model	Associated Product/Platform	Development Stage	Year Introduced	Clinical Benefit	Key References
**Prostate MRI lesion detection & segmentation**	CNN (deep learning), Radiomics	Research tools (no single commercial product)	Preclinical/Research	2017	Improved lesion detection, reduced inter-reader variability, biopsy targeting support	[9,10,11,14,15,16,17,18]
**Renal mass classification (benign vs. malignant, subtyping)**	CNN (ResNet), Radiomics + ML	Research tools	Preclinical/Research	2019	Non-invasive tumor characterization, support for treatment planning	[19,20,21]
**Bladder cancer staging (NMIBC vs. MIBC)**	CNN, Hybrid DL-ML models, DLRN	Research tools	Preclinical/Research	2019	Non-invasive staging support, treatment triage	[22,23,24,25]
**Surgical skill assessment (video-based)**	CNN, RNN, Computer vision, Optical flow	VAST system, Proximie AR platform (research use)	Clinical feasibility/Pilot use	2019	Objective skill evaluation, personalized training feedback, accelerated learning	[2,26,27,28,29]
**AR-guided partial nephrectomy**	3D reconstruction + AR overlay, CNN (ResNet-50)	HA3D^®^ (Medics^®^); iKidney (research prototype)	Limited clinical deployment/Commercially available for HA3D; Preclinical/Research for iKidney	2018	Selective clamping, nephron-sparing, reduced ischemia, improved renal function preservation	[30,31,32,33,34,35,36]
**AR-guided radical prostatectomy**	mpMRI-based 3D models + AR overlay	HA3D^®^ (Medics^®^)	Limited clinical deployment	2018	Real-time ECE visualization, nerve-sparing guidance, improved anatomical orientation and support for nerve-sparing and margin-conscious dissection	[37,38,39,40,41]
**AI-enhanced margin guidance**	PSMA PET-CT analysis, MRI-derived tumor mapping	Research tools	Preclinical/Research	2022	High diagnostic accuracy (up to 97–99%), improved spatial orientation for margin control	[40,41]
**Immersive surgical planning (VR/Metaverse)**	VR + 3D virtual models, collaborative platforms	Experimental platforms	Clinical feasibility/Pilot use	2023	Collaborative preoperative planning, enhanced spatial understanding	[42]
**Single-port robotic surgery**	SP robotic platform	da Vinci SP (Intuitive Surgical)	Regulatory-cleared/Commercially available	2018	Reduced access trauma, faster recovery, same-day discharge feasibility, advantages in confined spaces	[7,43,44,45,46,47,48,49,50,51,52,53,54,55,56,57,58,59,60]
**da Vinci 5 platform**	Next-gen robotic system with enhanced data capture and computing	da Vinci 5 (Intuitive Surgical)	Regulatory-cleared/Commercially available	2024	Enhanced imaging, AI-ready data infrastructure, improved ergonomics, foundation for real-time AI	[61]
**Telesurgery (5G-enabled)**	5G remote robotic control, multi-console configurations	MicroHand Edge MP1000, Toumai^®^, KangDuo	Emerging/Limited clinical deployment	2020	Access to expert surgery in remote areas, real-time remote collaboration across vast distances	[8,62,63,64,65,66]

**Abbreviations:** AI = Artificial Intelligence; AR = Augmented Reality; AUC = Area Under the Curve; CNN = Convolutional Neural Network; CT = Computed Tomography; DL = Deep Learning; DLRN = Deep Learning Radiomics Nomogram; ECE = Extracapsular Extension; HA3D^®^ = Hyper Accuracy 3D; MIBC = Muscle-Invasive Bladder Cancer; ML = Machine Learning; mpMRI = Multiparametric Magnetic Resonance Imaging; NMIBC = Non-Muscle-Invasive Bladder Cancer; PET = Positron Emission Tomography; PSMA = Prostate-Specific Membrane Antigen; RNN = Recurrent Neural Network; SP = Single-Port; VAST = Video Analysis of Skill and Technique; VR = Virtual Reality.

## Data Availability

Data sharing is not applicable. No new data were created or analyzed in this study.
